# The novel miR-1269b-regulated protein SVEP1 induces hepatocellular carcinoma proliferation and metastasis likely through the PI3K/Akt pathway

**DOI:** 10.1038/s41419-020-2535-8

**Published:** 2020-05-05

**Authors:** Lu Chen, Dongming Liu, Xianfu Yi, Lisha Qi, Xiangdong Tian, Bo Sun, Qiuping Dong, Zhiqiang Han, Qiang Li, Tianqiang Song, Mingxi Guo, Wei Zhang, Hua Guo, Ti Zhang

**Affiliations:** 1 Department of Hepatobiliary Cancer, Liver Cancer Research Center, Tianjin Medical University Cancer Institute and Hospital, National Clinical Research Center for Cancer, Key Laboratory of Cancer Prevention and Therapy, Tianjin’s Clinical Research Center for Cancer, Tianjin, 300060 China; 2 Department of Tumor Cell Biology, Tianjin Medical University Cancer Institute and Hospital, Tianjin, 300060 China; 3 Department of Biomedical Engineering and Technology, Tianjin Medical University, Tianjin, 300060 China; 4 Department of Pathology, Tianjin Medical University Cancer Institute and Hospital, Tianjin, 300060 China; 5 Department of Endoscopy, Tianjin Medical University Cancer Institute and Hospital, Tianjin, 300060 China; 6 The Second Department of Breast Cancer, Tianjin Medical University Cancer Institute and Hospital, Tianjin, 300060 China; 7 Department of Veterinary Diagnostic Laboratory, Iowa State University, Ames, IA 50011 USA

**Keywords:** Gastrointestinal cancer, Tumour biomarkers, Cell adhesion

## Abstract

Decreased intercellular adhesion is a key step in the metastasis and recurrence of many cancers, including hepatocellular carcinoma (HCC). SVEP1 is an important cell adhesion molecule that plays a key role in regulating intercellular adhesion and embryonic lymphatic development. However, the expression patterns and roles of SVEP1 in HCC are still largely unknown. We identified SVEP1 expression by analyzing 220 HCC samples from our cancer center. TCGA and GEO online-databases were used for data calibration and validation. SVEP1 was differentially expressed in two groups of HCCs with different risks of recurrence and was deemed as an independent risk factor for the prognosis of HCC. The expression of SVEP1 is negatively related to the proliferation and metastasis of HCC. Downregulation of SVEP1 expression promoted in vitro HCC cell migration, chemotaxis, invasion and proliferation, as well as in vivo tumor growth, local invasion and metastasis in a mouse model. Bioinformatic analysis and RT-PCR results showed that miR-1269b expression is negatively correlated with the SVEP1 expression and the prognosis of HCC patients. Further experiments showed that miR-1269b directly targets and downregulates the expression of SVEP1, which further induces the phosphorylation of Akt at thr308. These regulatory effects ultimately mediate the proliferation and metastasis of HCC cells. SVEP1 could serve as a promising prognostic marker of HCC. MiR-1269b downregulates SVEP1 expression and promotes HCC proliferation and metastasis likely through the PI3k/Akt signaling pathway.

## Introduction

Primary liver cancer (PLC) is the second leading cause of cancer-related death worldwide. The US 2019 cancer statistics report shows that the incidence of liver cancer is the highest among the common malignant tumors of men and women^1^. Hepatocellular carcinoma (HCC) is the major type of PLC, accounting for ~80% of PLCs^2^. Surgical resection and liver transplantation are considered to be the best approaches for early-stage HCC treatment^3^. However, when it comes to intermediate stage-Barcelona Clinic Liver Cancer (BCLC) B stage HCC, it is difficult to determine which treatment is more appropriate. Some patients achieve better survival after surgical resection, while others experience recurrence and metastasis within a short period of time after surgery^4,5^. Short-term recurrence and metastasis after surgery is one of the main factors affecting the prognosis of patients with HCC, especially BCLC B stage patients^6^.

Tumor recurrence and metastasis is a complex process involving multiple steps^7^. In this process, reduced intercellular adhesion plays an important role in tumor invasion and metastasis^8^. Previous studies have shown that the degradation of cell adhesion molecules (CAMs) is closely associated with the decrease in tumor cell adhesion. The structural changes and abnormal expression of CAMs mediate the decrease of adhesion, which facilitates tumor cell detachment from the primary focus, invasion into the surrounding tissues, entry into the blood circulation, and eventual formation of distant metastases^9^. The study of CAMs showed that PDLIM1 inhibits colorectal cancer cell metastasis by stabilizing the E-cadherin/beta-catenin complex. The degradation of PDLIM1 directly promotes the invasion and metastasis of colorectal cancer cells^10^. Another study revealed that the downregulation of LIPC expression in HCC cells increases the expression of CLDN10 and CLDN1 (CAMs) inhibits the proliferation and metastasis of HCC cells^11^. Therefore, the exploration of CAMs will facilitate the further exploration of the relevant mechanisms of tumor metastasis and improve the survival rate of cancer patients.

*SVEP1* (also known as *POLYDOM*), is located on chromosome 9q32 and covers 214 KB of genomic DNA^12^. Previous studies have shown that SVEP1, an important CAM, plays a central role in regulating intercellular adhesion^13^. A study on septic shock showed that the downregulation of SVEP1 expression in vascular endothelial cells induces the release of chemokines such as IL-8, MCP-1, and MCP-3, which mediate the enhancement of cell chemotaxis^14^. The decreased expression of SVEP1 in keratinocytes correspondingly reduces the expression of epithelial markers and affects epithelial cell phenotypic differentiation. There are significant differences in adhesion between normal and low-SVEP1-expressing keratinocytes^15^. Moreover, Glait-Santar et al.^16^ reported that alternative splice forms of *SVEP1* and their regulation may play a role in cancer cell invasion within the bone niche. However, the function and mechanisms of SVEP1 in malignant tumor progression remain largely unknown.

In this study, we selected 9 BCLC B stage HCC patients with similar clinicopathological characteristics and divided them into two groups according to disease-free survival (DFS) differences. Then we analyzed the genes that were differentially expressed between two groups through high-throughput RNA sequencing. The results revealed that differentially expressed genes (DEGs) are significantly enriched in the cell adhesion signaling pathway and that the mRNA level of *SVEP1* is significantly different between the two groups. By using TCGA and GEO database validation and immunohistochemical (IHC) staining of tissue microarrays of 207 HCC cases, we confirmed that low SVEP1 expression is closely associated with the progression and metastasis of HCC. Further in vivo and in vitro experiments showed that knockdown of SVEP1 expression promotes the HCC invasion and metastasis. Molecular mechanism studies revealed that SVEP1 expression is negatively regulated by miR-1269b, which induces PI3K/Akt signaling pathway activation and mediates the recurrence and metastasis of HCC. Thus, SVEP1 might be a novel biomarker for HCC diagnosis and a promising HCC therapeutic target.

## Materials and methods

### Patients and tissue specimens

A total of 220 patients with HCC who underwent liver resection in Tianjin Medical University Cancer Institute and Hospital between January 2010 and December 2014 were included in this study. Patients who had palliative surgery only, trans-hepatic artery embolization, chemotherapy, or radiotherapy were excluded from the study. The board-certified pathologists examined all paraffin-embedded specimens using hematoxylin and eosin staining. All patients provided written informed consent before we obtained the samples that were used in this study. The Research Ethics Committee of Tianjin Medical University Cancer Institute and Hospital granted ethical approval for the use of human subjects (Approval No. bc2020007) and the study was consistent with the ethical guidelines of the Helsinki Declaration.

### Cell culture

Hep3B, PLC, and HEK293T cells were purchased from American Type Culture Collection (ATCC; Manassas, VA, USA). Huh7 and HLE cell were bought from the Health Science Research Resources Bank (Shanghai, China) and Health Science Research Resources Bank (Osaka, Japan), respectively. MHCCLM3, MHCC97H, and MHCC97L cells were donated by the Liver Cancer Institute of Zhongshan Hospital, Fudan University. The cell lines were cultured in complete medium DMEM supplemented with 10% fetal bovine serum (FBS; PAN-Seratech) and 1% penicillin-streptomycin solution (PS; HyClone) under culture requirements (37°C; 5% CO_2_).

### mRNA sequencing analysis

150 bp paired-end reads were checked for the quality using FastQC (v0.11.8). Then Salmon (0.8.0) was used for quantification estimation based on gene annotation for human build hg38 downloaded from GENCODE (release 28). Differential gene expression was analyzed by DESeq2 based on Salmon quantification results and gene annotation. DEGs were filtered by log2 (Fold Change) ≥1 and adjust *p* value ≤ 0.05. The DEGs unique to high recurrent group were identified by two steps. First, the DEGs were identified for the high recurrent group and the low recurrent group, respectively, by comparing the tumor tissues to the corresponding adjacent normal tissues. Second, only the DEGs which identified in the high recurrent group but not in the low recurrent group were selected as the DEGs and used for the following analysis. ClusterProfiler was used for gene enrichment analysis of gene ontology and KEGG, and FDR ≤0.05 was used as the enrichment cutoff. To construct the interaction network, protein-protein interaction data from STRING database was used. The RNA-seq data used and/or analyzed during this study are available from the corresponding author on reasonable request.

### Transfection assay

Transfections were performed with the Lenti-Pac HIV Expression Packaging Kit (GeneCopoeia, Rockville, MD, USA). For generating lentiviral particles, packaging plasmids (VSVG and ΔR) and expression plasmids (KD and KD/SCR) were transfected into HEK293T cells using Lipofectamine 2000 (Invitrogen). The cells are then infected with a lentivirus to produce stable SVEP1 KD or KD/SCR cells. Six-well plates placed with 2 × 10^5^ cells were infected by lentivirus for 6–8 h. Then, added equal volume of 10% FBS DMEM and the transfected cells were cultured for another 48 h. The stably transfected cell line was obtained under the puromycin (GIBCO) selection about 7 days.

### Immunohistochemistry

IHC staining was used to detect the expression level of SVEP1 in paraffin samples from HCC and normal tissues. The final IHC score of SVEP1 was the product of the staining intensity score and the percentage score. Final staining score ≥2 was defined as SVEP1 high expression; final staining score <2 was defined as SVEP1 low expression. IHC scoring rules are as following: IHC staining intensity was evaluated in 4 classes (0, no immune response; 1 weak immune response; 2 medium-strong immune response; 3 strong immune response). The percentage of staining of immunohistochemistry was also classified into 4 scales (0, no positive cells; 1, <30% positive cells; 2, 30–60% positive cells; and 3, 60–100% positive cells).

### RNA extraction, cDNA synthesis, and quantitative real-time PCR

Total RNA was isolated from adherent cells using TRIzol reagent (Ambion, USA). cDNA was synthesized by reverse transcription of RNA using a quantitative RT-PCR kit (Takara, Japan). The amplification reaction was carried out using pre-designed primers according to the manufacturer’s instructions (Takara, Japan) with a Bio-Rad CFX96 system. For miRNA quantification, according to the manufacturer’s instructions, miScript PCR system (QIAGEN) were used and reverse-transcription RT-PCR was performed. U6 small nuclear RNA (RNU6B, QIAGEN) and GAPDH served as endogenous controls.

The amplification reaction for cDNA detection was carried out for 40 cycles. Each cycle contained denaturation at 95°C for 30 s, annealing for 5 s, and an extension at 60°C for 30 s. About miRNA detection, PCR initial activation step was set for 15 min at 95°C. Then the amplification reaction repeated 40 cycles. Each cycle contained denaturation at 94°C for 15 s, annealing at 55°C for 30 s, and an extension at 70°C for 30 s. The primers sequences were shown in Table S1.

### Western blotting of HCC tissues and cells

The fresh frozen HCC tissues were extracted with a tissue protein extractor. Western Blot was used to compare the SVEP1 expression level in tumor and para-tumor tissue. The cells were washed 3 times with cold PBS and lysed on ice for 30 min using SDS lysis buffer supplemented with 1 mM NaF, 1 mM Na3VO4, and 1x protease/phosphatase inhibitor cocktail. (Roche, Switzerland). The collected proteins were denatured at 95°C for 10 min and centrifuged at 12,000 rpm at 4°C about 10 min. Equal amounts of protein were loaded onto the gel and separated by SDS-PAGE. Then, transfer the protein to the PVDF membrane (Immobilon-P; Millipore, Billerica, MA, USA) and blocked with 5% skimmed milk or 3% bovine serum albumin (BSA), followed by incubation with primary and secondary antibodies. The following antibodies were used: anti-SVEP1 (1:250) from R&D SYSTEM; anti-GAPDH (1:1000) from Santa Cruz Biotechnology (Santa Cruz, CA, USA); anti-Akt (1:1000), anti-Phospho-Akt (Thr308) (1:2000) and Phospho-Akt (Ser473) (1:2000) from Cell Signaling Technology (Beverly, USA)

### Cell proliferation assay

The cells grew in 96-well plates at a density of 2 × 10^3^ cells per well for initial concentration. Five parallel wells were set in each group, as well as the blank control wells. Then, the cells were incubated with Cell Counting Kit-8 (Dojindo Laboratories, Kumamoto, Japan; 10 μL/well) for 4 h at 37°C and 5% CO_2_. The OD value of the wavelength at 450 nm was measured by an enzyme labeling instrument. The cell proliferation curve was drawn by continuous detection for 4–5 days. We compared the OD value of the two groups on the last day by paired *t* test statistical analysis.

### Dynamic monitoring of cell movement by high-throughput microscopy

The cells were plated in six-well plates at a density of (1.5–2) × 10^4^ cells/mL and grown overnight. Then, they were placed in a high-content imaging and analysis system (HCS) for dynamic analysis and observation. About 18 h after plating, the data points were recorded every 0.5 h and 36 data points were totally collected. Harmony software was used to track the individual cell displacement and mean migration distance in each well to compare the motor ability of different SVEP1 expression levels.

### Wound healing assay

For the wound healing assay, 1.5–2 × 10^6^ cells were plated in a 6-well plate. The next day, a 10 μL pipette tip was used to generate an even wound in the petri dishes. After the petri dishes were washed with PBS twice, 2% FBS was added in DMEM medium. The distance of the wound was recorded and calculated by every 3 h, and 6 random distances were finally counted. Images were captured with a microscope at 10× magnification. Data processing was performed after 24–48 h recording, and Graph Pad was used to analyse the distance of two groups on the last time point such as 24 h or 48 h by paired *t* test and map the data.

### Chemotaxis and invasion assay

For the chemotaxis and invasion assay, 24-well plates and 8-μm-pore chemotaxis chambers (Falcon) were used. Especially, for the invasion assay, matrigel was thawed in the ice bath under aseptic conditions, diluted to a concentration of 1 mg/mL with PBS, and frozen at −20°C for further use. Then diluted matrigel was taken out, thawed in the ice bath, plated on the polycarbonate membrane of the 24-well plate transwell chamber with a volume of 50 μL, and polymerized into a gel at 37°C for 1 h. Then, 20% FBS medium was added to the bottom plate of the 24-well plate (600 μL /well), while 200 μL of the appropriate concentration of cell suspension was added to the chamber. After the incubating of the well plate containing the chambers was completed, the cells which failed to pass through the chamber were rinsed out. Then the cells were processed with PFA solution, 100% methanol solution and three-step staining kit. Three randomly selected fields under the upright microscope were imaged, and the number of cells passing through each field was counted for statistical analysis.

### Dual-luciferase assay

The SVEP1 3′-UTR (untranslated region) and mutants were cloned into the pmiR-RB-Report™ h-SVEP1-WT and pmiR-RB-Report™ h-SVEP1-MUT (Ribobio). HEK293T cells were cotransfected with pmiR-SVEP1 wild-type (WT) or mutant plasmid and miR-1269b mimics or negative controls (NC). Luciferase activity was measured 24 h after transfection using the Dual-Glo^®^ Luciferase Assay System (Promega) according to the manufacturer’s instructions.

### miRNA plasmids transfection assays

The miRNA mimics and inhibitors used in this study, as well as the scrambled negative control miRNAs, were purchased from RIBOBIO (Guangzhou, China). In each well of a six-well plate, 50 nM miRNA mimics or inhibitors were added or the corresponding NC were added. For plasmid transfection, 8 μL of ViaFectTM Transfection Reagent (Promega USA) was added to 2 μg of plasmid.

### miRNA sequencing analysis

miRNA data obtained from next generation sequencing was processed by sRNAbench (version 1.5). Then DESeq2 was used to get the differentially expressed miRNAs based on the threshold of log2 (Fold Change) ≥1 and adjust *p* value ≤ 0.05. The differentially expressed miRNAs unique to high recurrent group were identified by two steps. First, the differentially expressed miRNAs were identified for the high recurrent group and the low recurrent group, respectively, by comparing the tumor tissues to the corresponding adjacent normal tissues. Second, only the differentially expressed miRNAs which identified in the high recurrent group but not in the low recurrent group were selected as the differentially expressed miRNAs and used for the following analysis. The validated miRNA-target interactions from miRWalk2.0 were used for miRNA-target identification. By combining validated miRNA-target interactions from miRWalk2.0 and protein-protein interactions from STRING database, only the differentially expressed miRNAs which were related to SVEP1 were displayed in the interactions network.

### Mouse xenograft tumor model

For the subcutaneous tumor xenograft assay, 4 weeks old male NOD-SCID nude mice were purchased from the Nanjing Biomedical Research Institute of Nanjing University. The mice were randomly assigned to the experimental group and the control group. No blinding was done during the experiments. The suspension of SVEP1 down-expressed HCC cell line and the control cell line were made and washed 3 times with PBS, then resuspended, and the number of the cells was counted. The mice were used for the assay, and 5 × 10^6^ tumor cells at the volume of 100 μL were injected to the subcutaneous tissue of each mice by a 1 mL injector. The status of the mice was observed every 2 days. The tumor volume was checked with a Vern ier caliper, and the variation of weight of the mice was recorded by a scale. All animal work procedures were approved by the Ethics Committee of the Tianjin Medical University Cancer Institute and Hospital, China.

### **T**CGA and GEO datasets

We downloaded the raw data of TCGA (NATIONAL CANCER INSTITUTE) and GEO (NCBI) related to HCC on the official website. Then the data was normalized by R Studio. We analyzed the *SVEP1* and miR-1269b in matched tumor and para-tumor for TCGA database. Then, we further analysis the relationship of *SVEP1* and miR-1269b with the prognosis of HCC patients. The GEO database (GSE45436) was also used to verify the differences of *SVEP1* between tumor and para-tumor for HCC patients.

### Gene set enrichment analysis

GSEA was performed to determine whether the *SVEP1* mRNA level is related to biological states, including metastasis, proliferation and the low expression of some genes in tumor, on the basis of GSE88402 data sets for HCC using GSEA 4.0.0 (The Broad Institute of MIT and Harvard).

### Statistical analyses

SPSS 25.0 for Windows (SPSS Inc., Chicago, IL) was used to evaluate the data. The univariate Kaplan–Meier method and multivariate Cox method were used to analyze the independent risk factors and survival curve of HCC patients. Spearman were used to analyze the correlation between the score of SVEP1 and clinicopathological factors. Unpaired *t* test was used to analyze the expression of SVEP1 in tumor and para-tumor tissues of HCC patients.

## Results

### SVEP1 expression pattern and its correlation with prognosis in human HCC

**Fig. 1:**
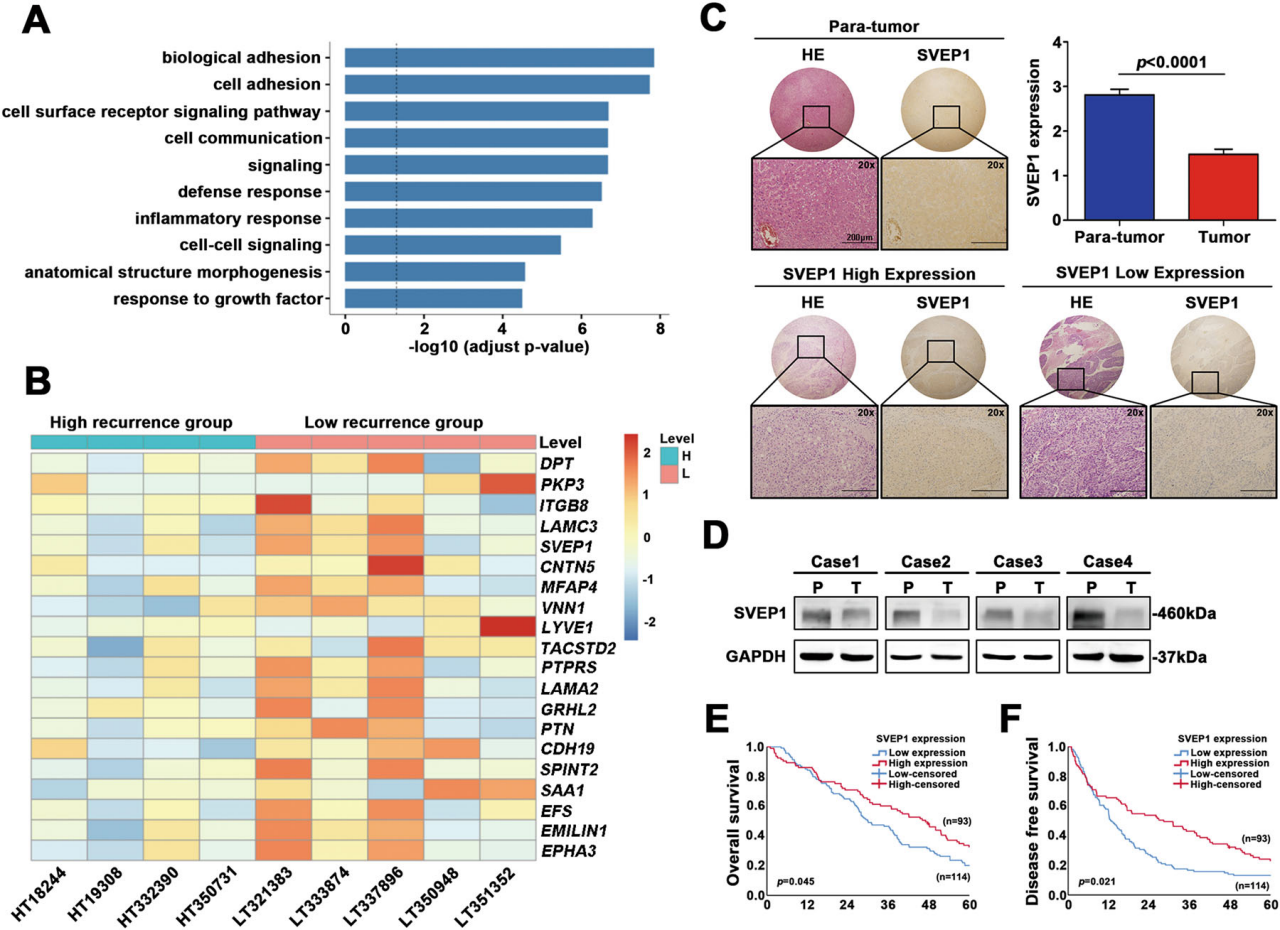
SVEP1 expression pattern and its correlation with prognosis in human HCC. **a**. Full transcriptome sequencing of two groups of HCC patients with different risk of recurrence; revealed the signaling pathways in which DEGs are enriched. **b** Heatmap of the top 20 DEGs in these two groups. **c** IHC and HE staining of SVEP1 in HCC and para-tumor tissues (the images shown are representative, the IHC scores of para-tumor, SVEP1 high expression tumor and SVEP1 low expression tumor are 6, 3, and 1, respectively). **d**. WB showing the differences in SVEP1 protein levels between four pairs of HCC tissues and para-tumor tissues. **e, f**. Kaplan–Meier survival curve analysis of the correlation between SVEP1 IHC scores and HCC patient OS and DFS (*n* = 207).

We collected nine BCLC B stage HCC samples with similar clinicopathological features (shown in Table S2). According to different DFS times, we divided these patients into two groups, the high-recurrence group (four cases, DFS <6 months) and the low-recurrence group (five cases, DFS >24 months; Table S2). Then, high-throughput RNA sequencing was applied to these clinical samples. By using DESeq2 analysis, a total of 1361 differentially expressed mRNAs were identified with the thresholds of a *p* value ≤ 0.05 and a | log2 FC | ≥ 1, including 787 mRNAs with upregulated expression and 574 mRNAs with downregulated expression. Notably, a signal pathway enrichment analysis based on these DEGs revealed that genes in the biological adhesion and cell adhesion signaling pathways were significantly enriched (Fig. 1a). Furthermore, the top20 DEGs in the adhesion-related pathways are shown in Fig. 1b. Strikingly, we found that *DPT*^17^, *PKP3*^18^ and *ITGB8*^19^ were all known to be correlated with tumor progression and metastasis. However, another remarkable DEG, *SVEP1*, was identified, and its role in cancer metastasis has never been explored (Fig. 1b).

To further explore the expression pattern of SVEP1 in HCC, a larger cohort of HCC samples including 207 paired tumor tissues and para-tumor tissues was determined by IHC. The results confirmed that SVEP1 expression was dramatically downregulated in tumor tissues compared with para-tumor tissues (*p* < 0.0001), and 114 out of 207 tumor tissues (55.07%) had low SVEP1 expression levels (Fig. 1c, Table S3). Next, we chose four pairs of HCC samples to subject to western blot (WB) and verified that the SVEP1 expression in the HCC tissues was substantially lower than that in the para-tumor tissues (Fig. 1d).

Then, the Kaplan–Meier method and Cox regression model were applied to investigate the correlation of SVEP1 expression with the prognosis of HCC patients. The multivariate analysis showed that elevated serum alpha-fetoprotein levels (≥20 ng/ml) and decreased expression of SVEP1 in tumor tissues were two independent risk factors for the prognosis of HCC (Table S3). The patients with low-SVEP1 expression tumors had a median overall survival (OS) and DFS time of 20.2 and 13.2 months, respectively, which was much worse than the median OS and DFS of 32.6 and 23.0 months for patients with high-SVEP1 expression tumors (*p* = 0.045, *p* = 0.021, Fig. 1e and f and Table S3). Consistently, the proportion of HCCs with low SVEP1 expression was significantly higher in the poor prognosis subgroup when compared with better prognosis subgroup (*p* = 0.002, Fig. S1). Thus, our data clearly indicate that SVEP1 is expressed at low levels in HCC tissues, and low SVEP1 expression levels correlate with a poor prognosis. We reasoned that decreased SVEP1 expression might play an important role in HCC progression and recurrence.

### The relationship between SVEP1 expression and clinicopathological features of HCC

**Fig. 2:**
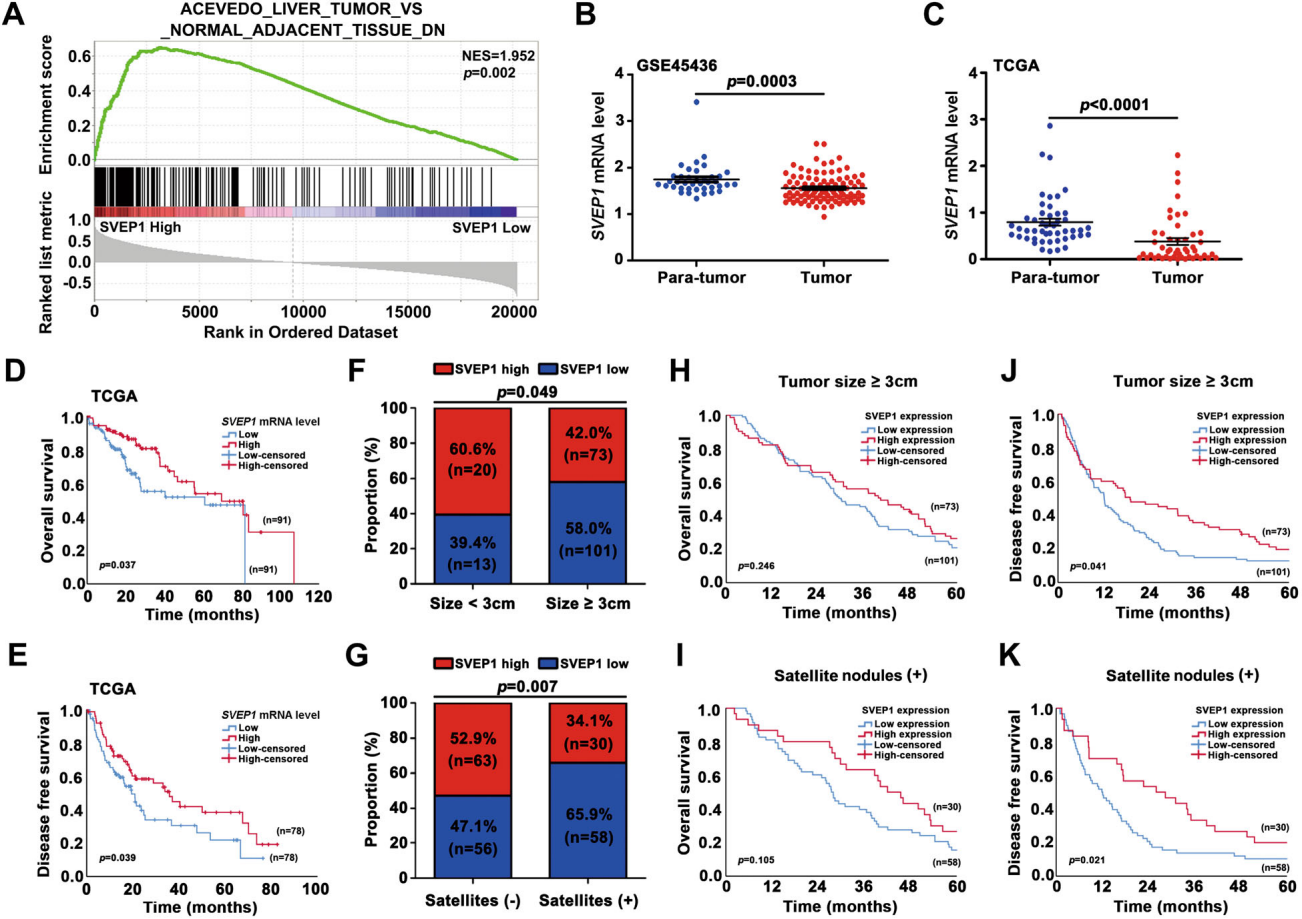
The relationship between SVEP1 expression and clinicopathological features of HCC. **a** GSEA validating the correlation between SVEP1 expression and the downregulated expression of genes in HCC tissues. **b, c**. GSE45436 and TCGA validation of the expression of SVEP1 in HCC and para-tumor tissues. **d, e** Kaplan–Meier survival curve of TCGA data to validate the correlation between the expression of SVEP1 and HCC patient OS and DFSs. **f, g**. Correlation analysis between the SVEP1 IHC score and clinicopathological parameters (tumor size and satellite nodules). **h, i**. Kaplan–Meier survival curve showing the correlation between the SVEP1 IHC score and the OS of a subgroup of HCC patients tumor size ≥3 cm and satellite nodule positive patients. **j**, **k** Kaplan–Meier survival curve showing the correlation between the SVEP1 IHC score and DFS of a subgroup of HCC patients (tumor size ≥3 cm and satellite nodules positive patients).

To further validate our previous research, we next performed a gene set enrichment analysis (GSEA) based on the mRNA data of HCC samples from GSE88402. The results confirmed that the level of *SVEP1* was downregulated in HCC tissues compared with corresponding adjacent tissues (*p* = 0.002, Fig. 2a). Moreover, similar results were also observed in the GSE45436 and TCGA database validation data (*p* = 0.003, *p* < 0.0001, Fig. 2b and c). In addition, based on the TCGA dataset, the relationship between *SVEP1* and OS in 360 HCC patients and DFS in 310 HCC patients were further analyzed. The results revealed that the mRNA level of *SVEP1* was positively correlated with HCC prognosis, by both OS and DFS (*p* = 0.037, *p* = 0.039, respectively, Fig. 2d and e).

Next, the associations of SVEP1 expression with various clinicopathological parameters were evaluated in our 207-patient cohort. We found that the expression level of SVEP1 was closely correlated with tumor size and satellite nodule occurrence (*p* = 0.049, *p* = 0.007, Table S4). Notably, tumor size and satellite nodules were the most critical independent risk factors for poor prognosis and the early recurrence of HCC^20^. A total of 101 out of 174 (58.0%) cases with a tumor diameter ≥3 cm had low SVEP1 expression levels. In contrast, only 13 out of 33 (39.4%) cases with a tumor diameter <3 cm had low SVEP1 expression levels (*p* = 0.049, Fig. 2f, Table S4). Furthermore, among the 88 HCC patients with satellite nodules, 58 (65.9%) had low SVEP1 expression levels, however, in HCC patients without satellite nodules, the percentage with low SVEP1 expression was only 47.1% (56/119; *p* = 0.007, Fig. 2g, Table S4).

Then, we further investigated the differential prognosis between high-SVEP1 expression HCCs and low-SVEP1 expression level HCCs in two high-risk relapse subgroups: tumor diameter ≥3 cm and tumor with satellite nodules. Surprisingly, in these two subgroups, although there was no significant difference in OS (*p* = 0.246, *p* = 0.105, Fig. 2h and i); patients with high levels of SVEP1 expression still had a longer DFS time than patients with low-SVEP1 expression levels (*p* = 0.041, *p* = 0.021, Fig. 2j and k). Taken together, these results suggest that a low level of SVEP1 expression is a critical feature of HCC that might pave the way for tumor proliferation and metastasis.

### Knock down of SVEP1 promotes HCC cell migration, invasion and proliferation in vitro

**Fig. 3:**
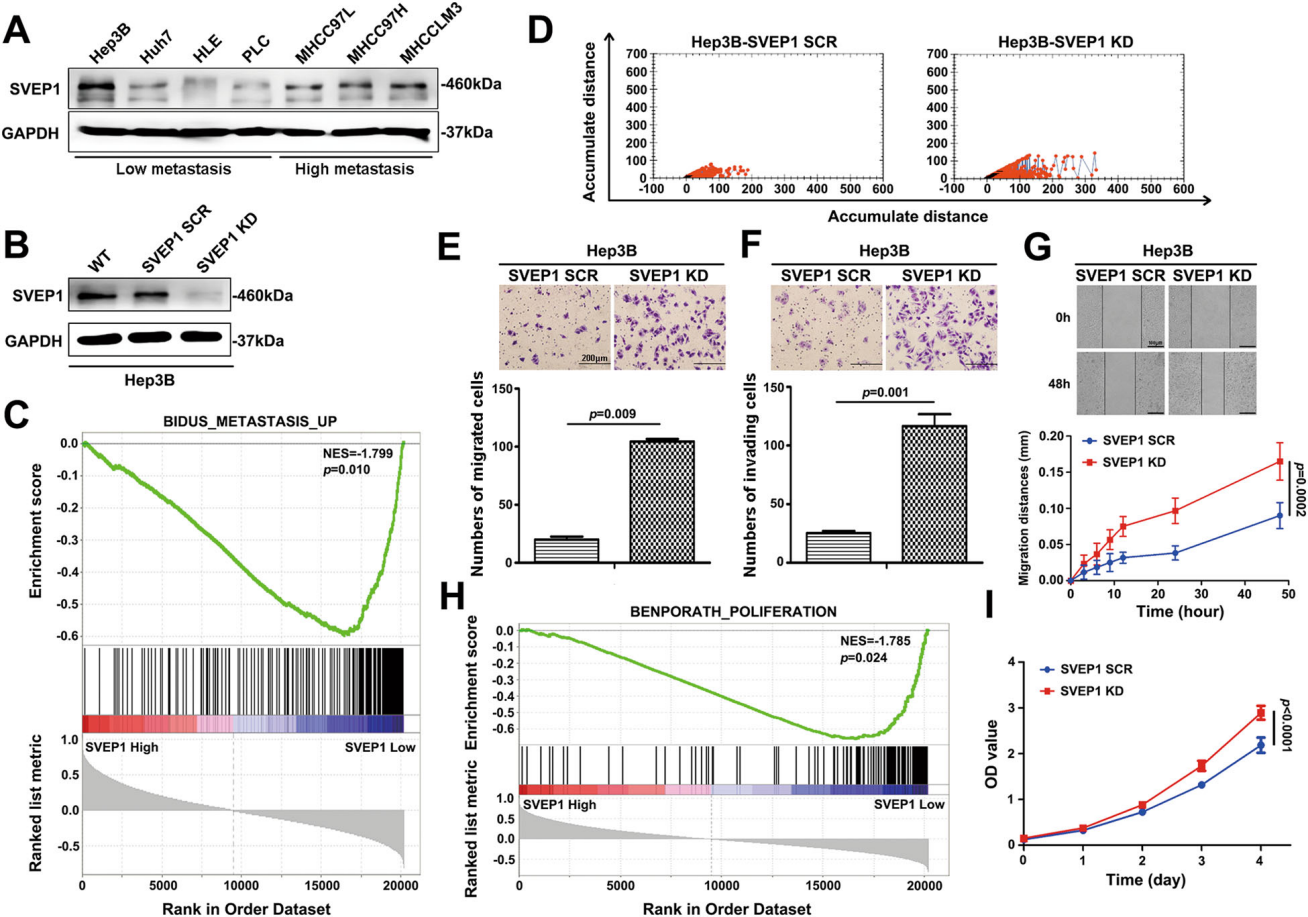
Knock down of SVEP1 promotes HCC cell migration, invasion, and proliferation in vitro. **a** WB showing the expression of SVEP1 at the protein level in 7 HCC cell lines (Hep3B, PLC, MHCC97L, MHCC97H, and MHCCLM3 are hepatitis B positive, Huh7 and HLE are hepatitis B negative). **b** The construction of the SVEP1 control group and SVEP1 knockdown Hep3B HCC cell line. **c** GSEA of the relationship between the expression of SVEP1 and tumor metastasis. **d** High-throughput microscopy comparing the migration ability of the SCR and SVEP1/KD groups. **e** Comparison of the chemotaxis potential the SVEP1/KD and SCR groups. **f** The invasive ability of the SVEP1/KD and SCR groups in the invasion assay. **g** Wound-healing assay comparing the translocation distance between the SVEP1/KD and SCR groups. **h** GSEA of the correlation between the expression level of SVEP1 and tumor proliferation. **i** CCK-8 assay showing the proliferation ability of the SVEP1/KD and SCR groups.

Next, we investigated the biological functions of SVEP1 in HCC proliferation and metastasis. First, we detected SVEP1 expression in HCC cell lines with different malignant potential and found that SVEP1 expression was the highest in Hep3B, which is known as a low-grade malignant tumor cell line (Fig. 3a). Hep3B and MHCCLM3 cells were used to establish stable knockdown cell lines, and the efficiency of SVEP1 deletion was confirmed by WB (Fig. 3b, Fig. S2A). A GSEA was then performed to investigate the biological role of *SVEP1* in HCC metastasis based on mRNA data from GSE88402. The data indicated that *SVEP1* was negatively associated with metastasis (Fig. 3c). Functional cell movement assay in Hep3B (Fig. 3d) and chemotaxis assay (Fig. 3e, Fig. S2B), transwell assay (Fig. 3f, Fig. S2C) and wound-healing assay (Fig. 3g, Fig. S2D) in both Hep3B and MHCCLM3 cells showed that SVEP1 knockdown promoted migration and invasion in vitro. In addition, the GSEA plot also suggested that low-*SVEP1* mRNA level was positively associated with proliferation (Fig. 3h). The results were further confirmed by a CCK-8 assay, which showed that SVEP1 expression downregulation significantly promoted HCC cell proliferation (*p* < 0.0001, Fig. 3i, Fig. S2e).

All these results suggest that the decrease in SVEP1 expression induces the metastasis and proliferation of HCC cells, which corroborates our previous clinical data analysis showing that patients with HCCs with low-SVEP1 expression levels have an increased likelihood of having increased tumors sizes and microsatellite nodules (Fig. 2f and g, Table S4).

### *SVEP1* is a direct target of miR-1269b

**Fig. 4:**
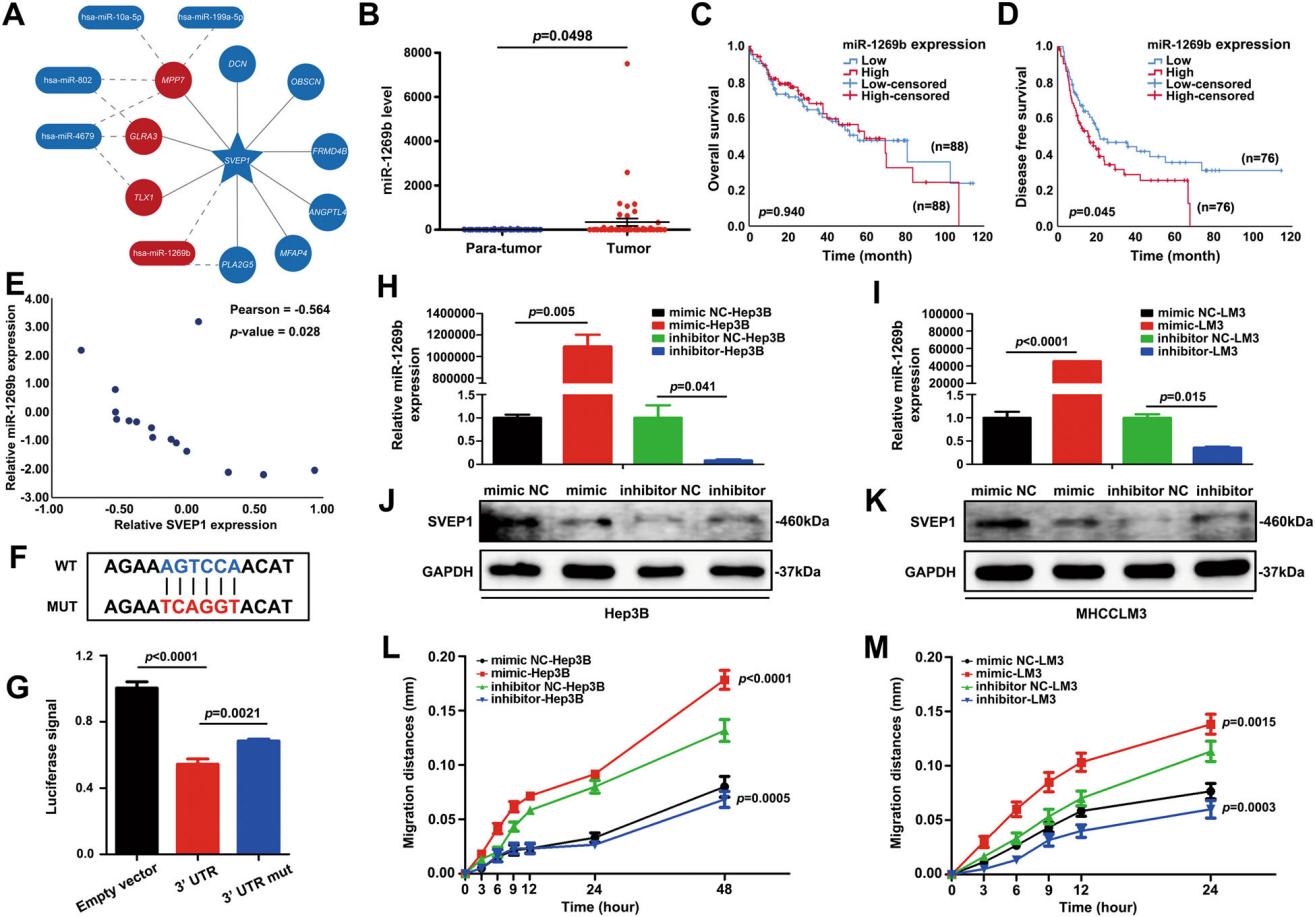
*SVEP1* is a direct target of miR-1269b. **a** Differentially expressed miRNAs in the high-throughput RNA sequencing. **b** The expression of miR-1269b in HCC tissues and para-tumor tissues in the TCGA database. **c, d** The correlation between the expression of miR-1269b and HCC patient OS/DFS in the TCGA miRNA database. **e** The correlation between the mRNA level of miR-1269b and *SVEP1* in HCC patients by RT-PCR. **f** The sequence of the 3′UTR of *SVEP1*. **g** Luciferase reporter assays of *SVEP1* and miR-1269b. **h, i** Establishment of different miR-1269b expression levels in Hep3B and MHCCLM3. **j, k** The verification of the expression level of the target gene (SVEP1) by WB in Hep3B and MHCCLM3. **l, m** Different migration abilities associated with different expression levels of miR-1269b in Hep3B and MHCCLM3 cells.

We further investigated the molecular mechanism by which SVEP1-mediates HCC proliferation and metastasis. First, we analyzed differentially expressed microRNAs (miRNAs) by using our high-throughput RNA sequencing data from 9 BCLC B stage HCC samples with different DFS times. A total of six differentially expressed miRNAs were discovered with the thresholds of a *p* value ≤ 0.05 and a | log2 FC | ≥ 1 by using DESeq2 analysis, including two miRNAs with upregulated expression and four miRNAs with downregulated expression (Fig. S3, Table S5 and S6). Next, miRNA-target interactions from miRWalk2.0 and protein-protein interaction networks from STRING were applied to analyze the differentially expressed miRNAs and mRNAs (identified in Fig. 1a) that might regulate or interact with *SVEP1*, respectively. Finally, miRNA-1269b was identified as a potential miRNA that might directly regulate the expression of SVEP1 (Fig. 4a).

MiR-1269b has been reported as an oncogene in HCC cells^21^. The overexpression of miR-1269b contributes to malignancy in HCC and might be a promising prognostic indicator for HCC patients^22^. However, the molecular mechanism of miR-1269b-induced HCC progression of HCC and its role in the regulation of SVEP1 expression remain largely unknown and need to be further clarified. We validated the expression of miR-1269b and its correlation with the prognosis of HCC patients in the TCGA database. The results showed that the expression level of miR-1269b was substantially higher in HCC tissues than in para-tumor tissues (*p* = 0.0498, Fig. 4b). Interestingly, although there was no significant difference in OS between high-miR-1269b expression and low-miR-1269b expression in HCCs (*p* = 0.940, Fig. 4c), patients with increased HCC miR-1269b expression always had a worse DFS time (*p* = 0.045, Fig. 4d). More importantly, the mRNA levels of miR-1269b and *SVEP1* in HCCs and their correlations were further confirmed in 15 HCC tissues by RT-PCR (Fig. 4e).

Moreover, to confirm the interaction between miR-1269b and the 3′UTR of *SVEP1*, we performed a luciferase reporter assay using luciferase reporters for WT *SVEP1* and 3′UTR-mutated *SVEP1* (85-90 AGTCCA > TCAGGT, Fig. 4f). As shown in Fig. 4g, miR-1269b efficiently reduced the luciferase activity of the *SVEP1*-3′UTR-WT reporter (*p* < 0.0001); however, the inhibition of luciferase activity by miR-1269b was partially reduced by the mutant *SVEP1* 3′UTR when compared with the WT *SVEP1*-3′UTR (*p* = 0.0021). To further verify the regulatory effect of miR-1269b on *SVEP1*, miR-1269b mimics/scrambled controls and inhibitors/scramble controls were transfected into Hep3B cells and MHCCLM3 cells, respectively (Fig. 4h and i). Strikingly, the expression level of SVEP1 in Hep3B and MHCCLM3 cells was reduced by miR-1269b mimic transfection and increased by miR-1269b inhibitor transfection (Fig. 4j and k). Notably, miR-1269b overexpression increased HCC cell migration (*p* < 0.0001, *p* = 0.0015, Fig. 4l and m). On the contrary, the downregulation of miR-1269b expression suppressed the migration of Hep3B and MHCCLM3 cells (*p* = 0.0005, *p* = 0.0003, Fig. 4l and m). Taken together, these results suggest that miR-1269b targets *SVEP1* and regulates the expression of SVEP1 in HCC, which contributes to the malignant phenotypic transformation of HCC cells.

### SVEP1 knockdown promotes proliferation and metastasis through the PI3K/Akt signaling pathway in HCC

**Fig. 5:**
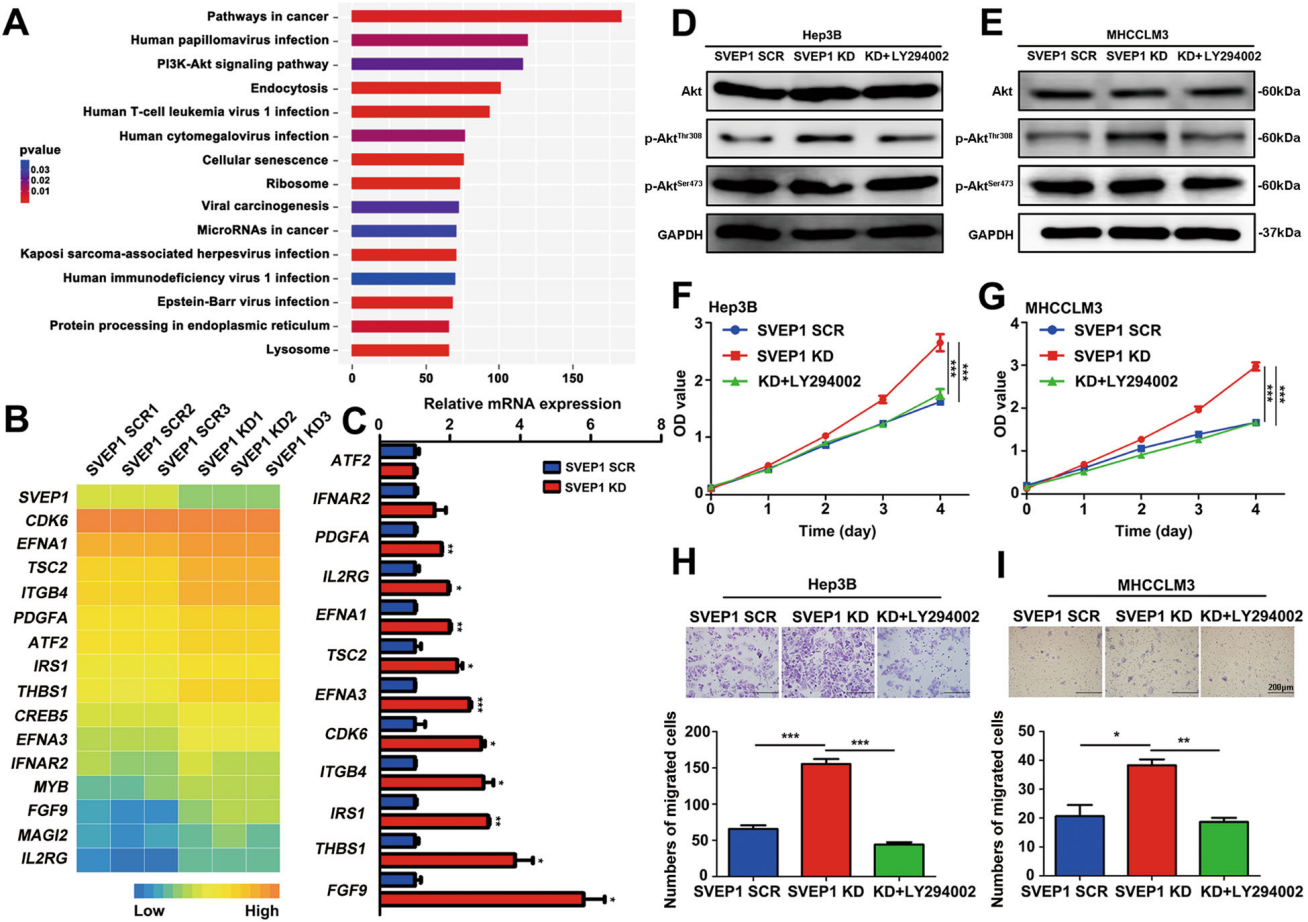
Knockdown of SVEP1 promotes proliferation and metastasis through the PI3K/Akt signaling pathway in HCC. **a** Signal pathway enrichment analysis of SCR and SVEP1/KD groups by RNA-seq in Hep3B cells. **b** Heatmap of 15 DEGs in the microarray. **c** The differential expression of the 15 DEGs in the SCR and SVEP1/KD groups by RT-PCR. **d**, **e** The effect of the downregulation of SVEP1 expression on PI3K/Akt signaling pathway activation was verified by WB in Hep3B and MHCCLM3 cells. **f**, **g** The effect of SVEP1 expression downregulation on proliferation in Hep3B and MHCCLM3 cells assessed by CCK-8 assay. **h, i** The effect of SVEP1 expression downregulation on chemotaxis in Hep3B and MHCCLM3 cells.

To further explore the downstream molecular mechanism of SVEP1, we conducted an RNA-seq analysis of Hep3B cells stably expressing a SVEP1 knockdown plasmid or empty vector. With the threshold of a *p* value < 0.05 and a | log2 FC | > 0.0, a total of 5578 DEGs were detected, including 2960 upregulated DEGs and 2618 downregulated DEGs. Then, signal pathway enrichment was analyzed based on these DEGs (Table S7). Notably, in addition to those in pathways involved in cancer, genes in the PI3K/Akt signaling pathway were also significantly enriched (Fig. 5a and Table S8). Moreover, most of the 15 DEGs enriched in the PI3K/Akt pathway were further validated by subsequent RT-PCR (Fig. 5b and c). It must be noted that a large number of previous studies, including ours, have shown that the activation of the PI3K/Akt signaling pathway plays a crucial role in mediating the progression and metastasis of various malignant tumors, such as liver cancer and breast cancer^23–25^.

Next, we used WBs to verify the effect of the downregulation of SVEP1 expression on PI3K/Akt signaling pathway activation. The results showed that that the downregulation of SVEP1 expression in Hep3B and MHCCLM3 cells did not affect the expression level of total Akt or the phosphorylation of Akt at Ser473; however, it obviously enhanced the phosphorylation of Akt at thr308 (Fig. 5d and e). The phosphorylation of Akt (thr308) is known to be very important for the proliferation and invasion of various malignant tumors^26,27^. We further investigated the role of p-Akt-thr308 in SVEP1-mediated HCC proliferation and metastasis. Hep3B SVEP1-knockdown and MHCCLM3 SVEP1-knockdown cells were cultured with an exogenous specific inhibitor of PI3K (LY294002), respectively. Interestingly, the increased level of p-Akt-thr308 was significantly reduced in LY294002-treated cells compared with untreated cells in both Hep3B and MHCCLM3 SVEP1-knockdown cell lines (Fig. 5d and e). More importantly, the inhibition of the PI3K/Akt signaling pathway by LY294002 counteracted the effect of SVEP1 knockdown on the proliferation (Fig. 5f and g) and migration (Fig. 5h and i) of Hep3B and MHCCLM3 cells.

Because downregulated SVEP1 expression increased p-Akt-thr308 in Hep3B and MHCCLM3 cells, we hypothesized that the phosphorylation of Akt at thr308 is the downstream molecular mechanism by which SVEP1 knock down induces proliferation and metastasis in HCC cells.

### Decreased SVEP1 expression promotes HCC cell proliferation and metastasis in vivo

**Fig. 6:**
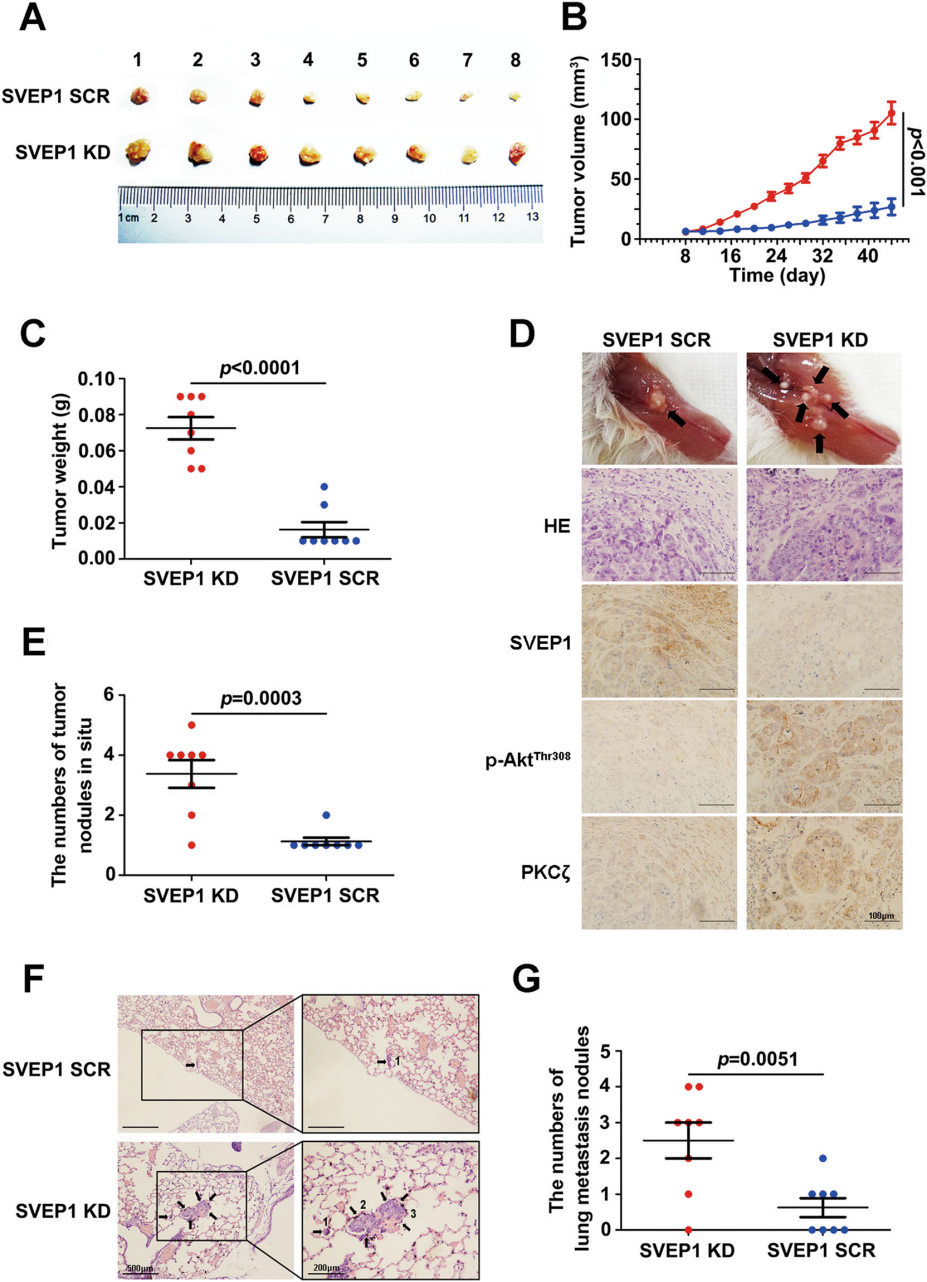
Decreased SVEP1 expression promotes HCC cell proliferation and metastasis in vivo. **a** Images of tumors from NOD-SCID nude mice in the SCR and SVEP1/KD groups. **b**, **c** The tumor volume and weight of the two groups. **d** HE and representative IHC staining of SVEP1, Akt^Thr308^ and PKCζ in tumor sections derived from SCR and SVEP1/KD HCC cell-derived models (the images shown are representative). **e** Statistical analyses of the bone invasion and multiple in situ metastases of the two groups. **f, g** Representative images and statistical analysis of the lung metastatic nodules in the two groups.

To further investigate the roles of SVEP1 in tumor growth and metastasis in vivo, Hep3B cells stably expressing SVEP1 shRNA or a control vector were subcutaneously implanted into NOD-SCID nude mice (5 × 10^6^/mouse). The volume of the tumors was measured every other day from the second week after the implantation. All mice in the two groups were sacrificed at the end of the 6th week, and the volume and weight of the primary tumors were evaluated. We found that compared with the control group, the SVEP1-knockdown group had dramatically accelerated tumor growth and markedly formed larger and heavier tumor nodules (*p* < 0.0001, Fig. 6a–c). Remarkably, along with the downregulation of SVEP1, the expression levels of p-Akt-thr308 and AKT target, PKCζ, were also decreased in SVEP1-knockdown group (Fig. 6d).

Then, we checked and analyzed the in situ invasion and distant metastasis of the tumors in two groups. Strikingly, most of the tumors formed in the SVEP1 knockdown group were accompanied by bone invasion and multiple in situ metastases; however, the tumors in the control group were noninvasive and single lesions (*p* = 0.0003, Fig. 6d and e). Consistently, the formation of metastatic lung nodules in the SVEP1 knockdown group was also markedly increased compared with that in the control group (*p* = 0.0051, Fig. 6f and g). Consistent with the findings in vitro, the results in vivo also demonstrate that suppression of SVEP1 in HCC cells promoted proliferation and metastasis likely though PI3K/Akt pathway.

## Discussion

HCC is a highly heterogeneous malignant solid tumor^2,28^. In China, more than 380,000 people die of liver cancer every year, accounting for 51% of global liver cancer deaths^29^. Surgical resection is still the primary curative choice for early-stage HCC^30^. Unfortunately, most patients with HCC in China are already in the middle (BCLC B) or advanced (BCLC C) stage at the time of diagnosis and miss the best optimal time for surgery^31^. In fact, for some BCLC B stage HCCs, surgical resection is still one of the best treatment choices. However, the effect of surgical resection varies greatly among different BCLC B stage patients. Some patients have a recurrence within a short period of time after surgery, whereas other patients have a long survival time.

We first compared the DEGs between two groups of BCLC B stage HCCs with different DFS time by high-throughput RNA sequencing, and found that abnormal variations in the adhesion-related pathway might play an important role in mediating the short-term recurrence of BCLC B stage HCCs after surgery. Our findings are consistent with those of previous studies showing that the reduction in cell-to-cell adhesion is a key process in the metastasis of cancer cells^32,33^. Therefore, into molecular mechanism that mediates the reduction in adhesion between HCC cells is necessary for the control of HCC recurrence.

It is noteworthy that by focusing on DEGs enriched in the adhesion-related pathway, we found a highly differentially expressed gene, *SVEP1*, which has not been previously reported in cancer. SVEP1 is a CAM and extracellular matrix protein that participates in lymphatic vessel remodeling and epidermal differentiation^15,34^. Significant associations between coronary artery disease and low-frequency missense variants in *SVEP1* have been discussed in previous studies^35^. Kakon Nag reported that a novel variant c.1159 G > T (p.Gly387Cys) in *SVEP1* may be as a critical genetic factor for liver cancer^36^. However, the role of *SVEP1* in cancer progression has never been reported before. In this study, we verified that the expression level of SVEP1 was significantly reduced in HCC tumor tissues and negatively correlated with HCC patient OS and DFS by using a large-scale HCC tissue microarray and bioinformatics analysis of online-public databases. Low SVEP1 expression is an independent risk factor for poor HCC prognosis. More importantly, the down-expression of SVEP1 in HCC cells significantly promotes the malignant transformation of HCC cells in vitro. The ability of tumor cells to move, chemotaxis, invade, and proliferate was significantly improved. Notably, further in vivo results suggested that the depression of SVEP1 expression in HCC cells accelerated the tumors growths, local skeletal invasion and distant lung metastasis. In summary, SVEP1 plays a vital role in mediating the proliferation and metastasis of HCC cells.

**Fig. 7:**
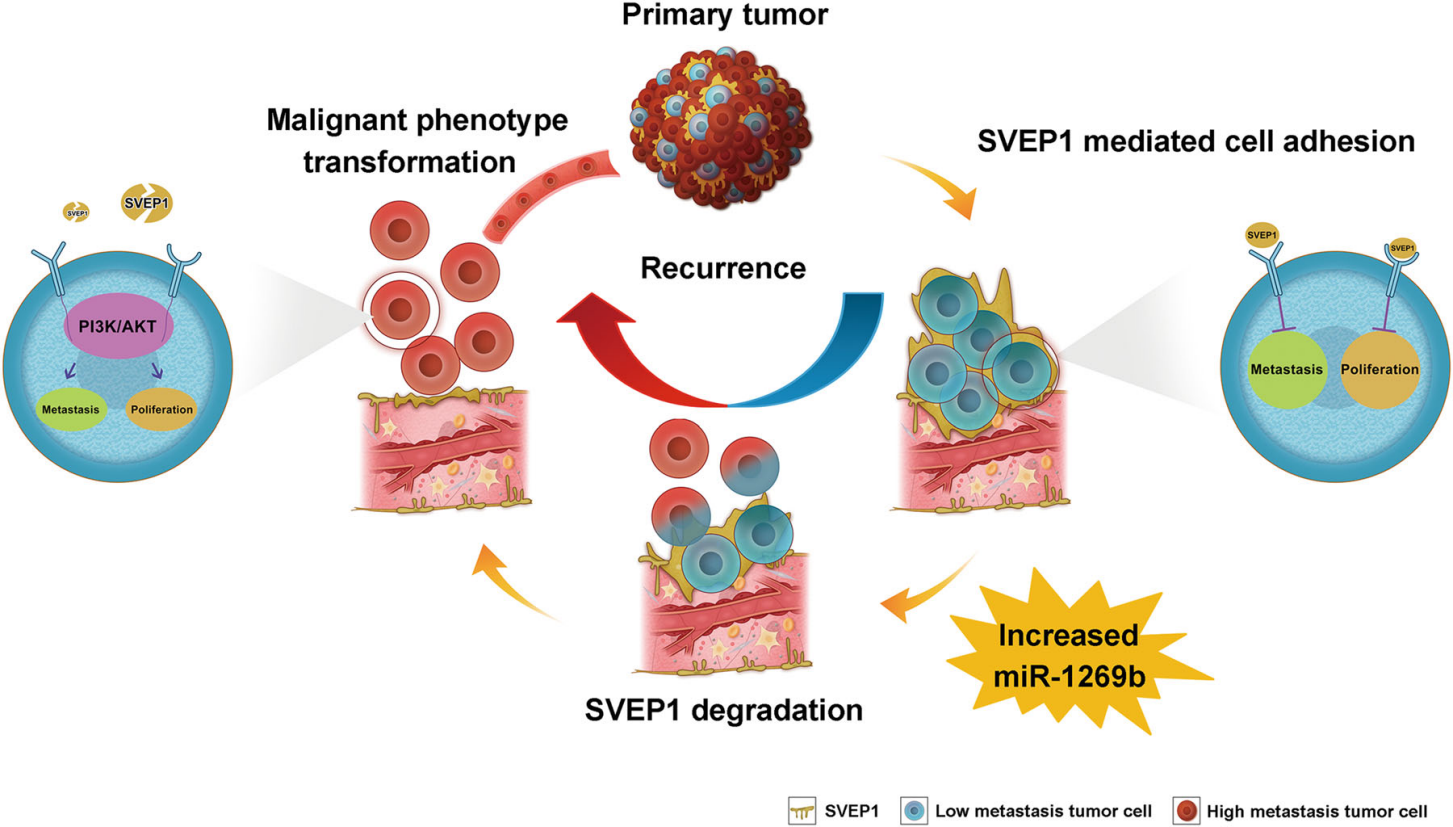
The proposed mechanistic scheme of SVEP1 induces proliferation and metastasis in HCC. SVEP1 is one of the most important cell adhesion molecules as well as extracellular matrix proteins that participates in cell-cell adhesion. There are several subclones with different characteristics in HCC. Some of them are highly expressing miR-1269b which is the key cause of SVEP1 degradation. Notably, decreased expression of SVEP1 in HCC further leads to the activation of PI3K/Akt signaling pathway, which may be the potential mechanism for the recurrence and metastasis of HCC.

MiRNAs are ~22-nt RNAs that are involved in the regulation of a range of cellular biological processes and the regulation of cancer^37^. MiR-1269b is considered to be an important cancer promoter in diverse cancer types. Previous studies have shown that miR-1269b genetic variants are biomarkers for the development of oral premalignant lesions and oral and pharyngeal squamous cell carcinoma^38^. Furthermore, the increased expression of CDC40 induced by mi-1269b is considered to be an important molecular mechanism underlying the progression of HBV-related HCCs^39^. Nevertheless, the biological effects of miR-1269b in HCC metastasis and recurrence still require in-depth exploration. Consistent with previous studies, we found that HCCs with high expression levels of miR-1269b were often associated with a reduced DFS time. More importantly, our data showed that the elevated expression of miR-1269b in HCCs could promote the malignant transformation of HCC cells by directly targeting *SVEP1* and inhibiting its expression. In contrast, reduced miR-1269b expression led to increased SVEP1 expression, which reversed the malignancy of HCC cells to some extent. Together, these findings suggest that the targeted regulation of SVEP1 expression by miR-1269b is a critical upstream molecular mechanism of the SVEP1-mediated progression of HCC (Fig. 7).

Another intriguing, but preliminary, observation from our study is that the DEGs induced by the downregulation of SVEP1 expression were mainly enriched in the PI3K/Akt signaling pathway. Activation of the PI3K/Akt signaling pathway is one of the most well-known molecular mechanisms of cancer progression^40^. Consistently, we found that the downregulation of SVEP1 expression in HCC cells induced the phosphorylation of Akt at thr308, which directly promotes the proliferation and metastasis of HCC cells. Moreover, the inhibition of the PI3K/Akt pathway mitigated the effect of SVEP1 knockdown on promoting the malignant transformation of HCC cells (Fig. 7). There are still several limitations in this part of the research. First, further molecular biological experiments are needed to explore the underlying mechanism of the PI3K/Akt signaling pathway activation induced by the downregulation of SVEP1. Second, because SVEP1 is a novel CAMs, previous studies on SVEP1 are very limited, which also restricted our exploration of the molecular mechanism to a certain extent. The phosphorylation of Akt at thr308 induced by the downregulation of SVEP1 expression was observed in our study. Thus, our findings might provide a basis for the exploration of the downstream mechanism of SVEP1 expression downregulation induced HCC progression in further studies.

In summary, we identified that low SVEP1 expression in patients with HCC is indicative of poor prognosis and early recurrence after surgery. Our results demonstrate that miR-1269b downregulates the expression of SVEP1 and activates the PI3k/Akt signaling pathway mediating HCC proliferation and metastasis in vitro and in vivo (Fig. 7). We hypothesize that SVEP1 could be used as a promising biomarker for the prediction of tumor progression and recurrence for the clinicopathological diagnosis of HCC.

## Supplementary information

Supplementary Figure Legends-Clean final word file

Supplementary figure 1

Supplementary figure 2

Supplementary figure 3

Supplementary Table Legends

Supplementary table 1

Supplementary table 2

Supplementary table 3

Supplementary table 4

Supplementary table 5

Supplementary table 6

Supplementary table 7

Supplementary table 8
