# Supplementary material for: The novel miR-1269b-regulated protein SVEP1 induces hepatocellular carcinoma proliferation and metastasis likely through the PI3K/Akt pathway
Source: Cell Death Dis. 2020 May 5;11(5):320. doi: 10.1038/s41419-020-2535-8 (PMC7200779; doi:10.1038/s41419-020-2535-8)
Supplement: Supplementary file 1 — Supplementary Figure Legends-Clean final word file [file 41419_2020_2535_MOESM1_ESM.docx]

**Supplementary Figure Legends**

**Figure S1. The ratio of low SVEP1 expression HCC patients in two different prognosis groups.** The ratio of low SVEP1 expression between the poor prognosis (DFS < 12 months) subgroup and better prognosis subgroup (DFS > 24 months) showed a significant difference.

**Figure S2. Knock down of SVEP1 promotes HCC cell migration, invasion and proliferation in MHCCLM3**

(A). Western blots analysis of the construction of the control group of SVEP1 and the down-expressed MHCCLM3 HCC cell line. (B). Chemotaxis potential of SVEP1 down-expressed group and control group compared by the Chemotaxis assay. (C). Invasive ability of down-expressed group and control group under microscope shown by the Invasion assay. (D). Migration distance of the down-expressed group and control group in the Wound healing assay. (E). Comparison of the proliferation ability of the down-expressed group and control group performed by the CCK8 assay.

**Figure S3. Six miRNAs including miR-1269b were identified in high recurrence group.** Compared with par-tumor or tumor-adjacent tissues, 6 miRNAs were uniquely differentially expressed in the high recurrence group, 5 miRNAs were uniquely differentially expressed in the low recurrence group, and 3 miRNAs were shared by both groups.
