# Supplementary material for: The novel miR-1269b-regulated protein SVEP1 induces hepatocellular carcinoma proliferation and metastasis likely through the PI3K/Akt pathway
Source: Cell Death Dis. 2020 May 5;11(5):320. doi: 10.1038/s41419-020-2535-8 (PMC7200779; doi:10.1038/s41419-020-2535-8)
Supplement: Supplementary file 5 — Supplementary Table Legends [file 41419_2020_2535_MOESM5_ESM.docx]

**Supplementary Table Legends**

**Table S1. RT-PCR primers of mRNA and miRNA.**

**Table S2. Clinicopathological factors of HCC patients for whole transcriptome sequencing (**Abbreviations: HBV, hepatitis B virus; AFP, alpha fetoprotein; Mavi, macrovascular invasion; Mivi, microvascular invasion).

**Table S3.** **Univariate and multivariate analysis of prognostic factors associated with OS and DFS in 207 HCC patients (**Final staining score ≥ 2 was defined as SVEP1 high expression; final staining score < 2 was defined as SVEP1 low expression. *: *p*<0.05 was considered statistically significant. Abbreviations: HBV, hepatitis B virus; Mavi, macrovascular invasion; Mivi, microvascular invasion; AFP, alpha fetoprotein; ALB, albumin; ALT, alanine aminotransferase; AST, aspartate aminotransferase; BCLC, Barcelona Clinic Liver Cancer).

**Table S4.** **Relationship between clinicopathological characteristics and SVEP1 expression in 207 HCC patients (**Final staining score ≥ 2 was defined as SVEP1 high expression; final staining score < 2 was defined as SVEP1 low expression. *: *p*<0.05 was considered statistically significant. Abbreviations: HBV, hepatitis B virus; Mavi, macrovascular invasion; Mivi, microvascular invasion; AFP, alpha fetoprotein; ALB, albumin; ALT, alanine aminotransferase; AST, aspartate aminotransferase; BCLC, Barcelona Clinic Liver Cancer**).**

**Table S5. miRNAs identified in high recurrence group.**

**Table S6. miRNAs identified in low recurrence group**

**Table S7.** **Signal pathway enrichment based on differentially expressed genes between Hep3B/SCR and Hep3B/SVEP1 KD cells.**

**Table S8. The raw data of differentially expressed genes in PI3K/Akt signal pathway.**
