# Supplementary material for: The novel miR-1269b-regulated protein SVEP1 induces hepatocellular carcinoma proliferation and metastasis likely through the PI3K/Akt pathway
Source: Cell Death Dis. 2020 May 5;11(5):320. doi: 10.1038/s41419-020-2535-8 (PMC7200779; doi:10.1038/s41419-020-2535-8)
Supplement: Supplementary file 6 — Supplementary table 1 [file 41419_2020_2535_MOESM6_ESM.docx]

| **Table S1. RT-PCR primers of mRNA and miRNA** | |
| --- | --- |
| **mRNA** | **primer sequences** |
| Human *ATF2* Forward Primer | AGTTGGGTAGATGCATCATGG |
| Human *ATF2* Reverse Primer | GGCATCCCCACCTTCCTTCTG |
| Human *CDK6* Forward Primer | CCCCAGAGTCTGATTACCTGC |
| Human *CDK6* Reverse Primer | ACATAGCCTCTGCCCAAGC |
| Human *CREB5* Forward Primer | ACTGAGGCAAATACTCAAGACT |
| Human *CREB5* Reverse Primer | GTTGGGAAGCGCTGGGA |
| Human *FGF9* Forward Primer | CCTGGTCAGCATTCGTGGTG |
| Human *FGF9* Reverse Primer | GCCGTTTAGTCCTGGTCCCT |
| Human *IRS1* Forward Primer | ACAAACGCTTCTTCGTACTGC |
| Human *IRS1* Reverse Primer | AGTCAGCCCGCTTGTTGATG |
| Human *ITGB4* Forward Primer | TTAAGAGAGCCGAGGAGGTG |
| Human *ITGB4* Reverse Primer | GGCAGTCCTTCTTCTTGTGC |
| Human *PDGFA* Forward Primer | GCAAGACCAGGACGGTCATTT |
| Human *PDGFA* Reverse Primer | GGCACTTGACACTGCTCGT |
| Human *THBS1* Forward Primer | AGACTCCGCATCGCAAAGG |
| Human *THBS1* Reverse Primer | TCACCACGTTGTTGTCAAGGG |
| Human *TSC2* Forward Primer | TCCTCGACCAGATCCCATCA |
| Human *TSC2* Reverse Primer | GCCATGCTCATTGGACAGGA |
| Human *MYB* Forward Primer | CACCCCATCTCTGCCTGCAGATCC |
| Human *MYB* Reverse Primer | CATGACCAGAGTTCGAGCTGAGAA |
| Human *EFNA3* Forward Primer | CTTGTGGCTCTGGTAATGTTTGG |
| Human *EFNA3* Reverse Primer | GAGGAGGACGTGCTTATTGCTGT |
| Human *EFNA1* Forward Primer | GTGGACATCATCTGTCCGCA |
| Human *EFNA1* Reverse Primer | GGACTTGGTCCTTGGACTGG |
| Human *IL2RG* Forward Primer | GGCTGAACACGACAATTCTGAC |
| Human *IL2RG* Reverse Primer | AATGCAGAGTGAGGTTGGTAGG |
| Human *MAGI2* Forward Primer | ATGGCTGGATCCACGACTTG |
| Human *MAGI2* Reverse Primer | GGTTTTTCTCGGAAACCTGGG |
| Human *IFNAR2* Forward Primer | GCGAGAGCTGCAAAGATGTAA |
| Human *IFNAR2* Reverse Primer | GGACCGGAAATTTCGCAATGA |
| Human *SVEP1* Forward Primer | GAGACCCCAGACCTATTGC |
| Human *SVEP1* Reverse Primer | ATATTCCCCTGCCAAATCCC |
| Human *GAPDH* Forward Primer | TGGTATCGTGGAAGGACTCA |
| Human *GAPDH* Reverse Primer | CCAGTAGAGGCAGGGATGAT |
| **miRNA** | |
| has-miR-1269b | 5'-CUGGACUGAGCCAUGCUACUGG-3' |
