# Supplementary material for: The novel miR-1269b-regulated protein SVEP1 induces hepatocellular carcinoma proliferation and metastasis likely through the PI3K/Akt pathway
Source: Cell Death Dis. 2020 May 5;11(5):320. doi: 10.1038/s41419-020-2535-8 (PMC7200779; doi:10.1038/s41419-020-2535-8)
Supplement: Supplementary file 7 — Supplementary table 2 [file 41419_2020_2535_MOESM7_ESM.docx]

| **Table S2. Clinicopathological factors of HCC patients for whole transcriptome sequencing** | | | | | | | | | | |
| --- | --- | --- | --- | --- | --- | --- | --- | --- | --- | --- |
| **Patient ID** | **Age** | **Sex** | **HBV** | **AFP (ng/mL)** | **Tumor size (cm)** | **Tumor number** | **Satellite nodule** | **Mavi** | **Mivi** | **Recurrence status** |
| HT18244 | 66 | Male | NA | 408.1 | 4*3*2 | 2 | (-) | (-) | (-) | High |
| HT19308 | 67 | Male | (-) | 933.8 | 4.2*3.8 | 2 | (-) | (-) | (-) | High |
| HT332390 | 59 | Male | (+) | 6.25 | 5*3*2 | 2 | (-) | (-) | (-) | High |
| HT350731 | 59 | Male | (+) | 320.1 | 6.5*4*3 | 2 | (-) | (+) | (-) | High |
| LT321383 | 49 | Male | (-) | 7808 | 5*5*4 | ＞3 | (+) | (+) | (-) | Low |
| LT333874 | 55 | Male | (-) | 3.94 | 2*2*1.5 | ＞3 | (+) | (+) | (-) | Low |
| LT337896 | 52 | Male | (-) | 2.21 | 4.5*4*4 | 2 | (-) | (+) | (-) | Low |
| LT350948 | 52 | Male | (+) | 65.45 | 5*4*4 | 3 | (-) | (-) | (-) | Low |
| LT351352 | 49 | Male | (+) | 113.4 | 7*7*5 | 2 | (+) | (+) | (-) | Low |
