# Supplementary material for: The novel miR-1269b-regulated protein SVEP1 induces hepatocellular carcinoma proliferation and metastasis likely through the PI3K/Akt pathway
Source: Cell Death Dis. 2020 May 5;11(5):320. doi: 10.1038/s41419-020-2535-8 (PMC7200779; doi:10.1038/s41419-020-2535-8)
Supplement: Supplementary file 8 — Supplementary table 3 [file 41419_2020_2535_MOESM8_ESM.docx]

| **Table S3.** **Univariate and multivariate analysis of prognostic factors associated with OS and DFS in 207 HCC patients** | | | | | | | | | |
| --- | --- | --- | --- | --- | --- | --- | --- | --- | --- |
| **HCC patients (n=207)** | **Number** | **Univariate Analysis** | | **Multivariate Analysis** | | **Univariate Analysis** | | **Multivariate Analysis** | |
|  |  | **5-year OS (%)** | ***p*-value** | **HR (95% CI)** | ***p*-value** | **5-year DFS (%)** | ***p-*value** | **HR (95% Cl)** | ***p*-value** |
| **Age(years) ≥55/<55** | 115/92 | 27.6/23.5 | 0.584 |  |  | 18.9/16.1 | 0.382 |  |  |
| **Sex male/female** | 166/41 | 27.3/19.5 | 0.283 |  |  | 19.0/12.2 | 0.544 |  |  |
| **HBV Y/N** | 161/46 | 24.6/29.7 | 0.151 |  |  | 15.4/25.9 | 0.310 |  |  |
| **Liver cirrhosis Y/N** | 117/90 | 30.2/20.0 | 0.081 |  |  | 19.3/15.6 | 0.353 |  |  |
| **Ascites Y/N** | 18/189 | 22.2/26.1 | 0.926 |  |  | 16.7/17.8 | 0.904 |  |  |
| **Blood transfusion in surgery Y/N** | 23/184 | 4.3/28.5 | **0.002*** | 1.536(0.936,2.520) | 0.089 | 4.3/19.4 | **0.023*** | 1.340(0.807,2.225) | 0.258 |
| **Tumor size(cm) ≥3/<3** | 174/33 | 23.2/39.4 | 0.052 |  |  | 15.8/27.3 | **0.023*** | 1.355(0.870,2.109) | 0.179 |
| **Mavi Y/N** | 22/185 | 9.1/27.7 | **0.001*** | 1.740(0.912,3.726) | 0.154 | 4.5/19.3 | **0.000*** | 2.023(0.963,4.250) | 0.063 |
| **Mivi Y/N** | 120/87 | 19.2/35.0 | **0.026*** | 1.253(0.903,1.740) | 0.177 | 15.8/20.4 | 0.140 |  |  |
| **Differentiation H/L** | 162/45 | 29.2/13.3 | **0.002*** | 1.219(0.818,1.816) | 0.330 | 19.5/11.1 | **0.011*** | 1.112(0.757,1.635) | 0.589 |
| **Satellite nodule Y/N** | 88/119 | 19.3/30.6 | 0.105 |  |  | 13.6/20.7 | 0.134 |  |  |
| **AFP (ng/mL) ≥20/<20** | 113/94 | 19.5/33.5 | **0.011*** | 1.399(1.006,1.946) | **0.046*** | 11.5/25.3 | **0.004*** | 1.535(1.113,2.119) | **0.009*** |
| **ALB (g/L) ≥40/<40** | 146/61 | 29.2/17.3 | 0.094 |  |  | 19.0/14.6 | 0.320 |  |  |
| **ALT (U/L) ≥50/<50** | 48/159 | 22.1/26.8 | 0.268 |  |  | 16.0/18.1 | 0.354 |  |  |
| **AST (U/L) ≥40/<40** | 69/138 | 17.4/30.0 | **0.004*** | 1.390(0.999,1.935) | 0.051 | 11.6/20.7 | **0.015*** | 1.277(0.917,1.779) | 0.148 |
| **P53 mutation Y/N** | 87/120 | 28.7/23.5 | 0.735 |  |  | 20.7/15.4 | 0.861 |  |  |
| **BCLC stage 0&A/B&C** | 172/35 | 29.3/8.6 | **0.000*** | 1.149(0.620,2.131) | 0.659 | 20.8/2.9 | **0.000*** | 1.055(0.555,2.006) | 0.869 |
| **SVEP1 High/Low** | 93/114 | 32.6/20.2 | **0.022*** | 0.723(0.526,0.993) | **0.045*** | 23.0/13.2 | **0.004*** | 0.690(0.504,0.945) | **0.021*** |
