# Supplementary material for: The novel miR-1269b-regulated protein SVEP1 induces hepatocellular carcinoma proliferation and metastasis likely through the PI3K/Akt pathway
Source: Cell Death Dis. 2020 May 5;11(5):320. doi: 10.1038/s41419-020-2535-8 (PMC7200779; doi:10.1038/s41419-020-2535-8)
Supplement: Supplementary file 9 — Supplementary table 4 [file 41419_2020_2535_MOESM9_ESM.docx]

**Table S4.** **Relationship between clinicopathological characteristics and SVEP1 expression in 207 HCC patients**

| **Characteristics** |  | **Total** | **SVEP1 expression** | | ***p*-value** | **Characteristics** |  | **Total** | **SVEP1 expression** | | ***p*-value** |
| --- | --- | --- | --- | --- | --- | --- | --- | --- | --- | --- | --- |
|  |  | **207** | **Low** | **High** |  |  |  | **207** | **Low** | **High** |  |
| **Age (years)** |  |  |  |  | 0.225 | **Differentiation** |  |  |  |  | 0.455 |
|  | ≥ 55 | 115 | 56 | 59 |  |  | High | 162 | 87 | 75 |  |
|  | < 55 | 92 | 55 | 37 |  |  | Low | 45 | 27 | 18 |  |
| **Sex** |  |  |  |  | 0.621 | **Satellite nodule** |  |  |  |  | **0.007*** |
|  | Male | 166 | 90 | 76 |  |  | Present | 88 | 58 | 30 |  |
|  | Female | 41 | 24 | 17 |  |  | Absent | 119 | 56 | 63 |  |
| **HBV** |  |  |  |  | 0.118 | **AFP (ng/mL)** |  |  |  |  | 0.622 |
|  | Present | 161 | 84 | 77 |  |  | ≥20 | 113 | 64 | 49 |  |
|  | Absent | 46 | 30 | 16 |  |  | <20 | 94 | 50 | 44 |  |
| **Liver cirrhosis** |  |  |  |  | 0.874 | **ALB (g/L)** |  |  |  |  | 0.856 |
|  | Present | 117 | 65 | 52 |  |  | ≥40 | 146 | 81 | 65 |  |
|  | Absent | 90 | 49 | 41 |  |  | <40 | 61 | 33 | 28 |  |
| **Ascites** |  |  |  |  | 0.345 | **ALT (U/L)** |  |  |  |  | 0.637 |
|  | Present | 18 | 8 | 10 |  |  | ≥50 | 48 | 25 | 23 |  |
|  | Absent | 189 | 106 | 83 |  |  | <50 | 159 | 89 | 70 |  |
| **Blood transfusion in surgery** |  |  |  |  | 0.768 | **AST (U/L)** |  |  |  |  | 0.768 |
|  | Present | 23 | 12 | 11 |  |  | ≥40 | 69 | 39 | 30 |  |
|  | Absent | 184 | 102 | 82 |  |  | <40 | 138 | 75 | 63 |  |
|  |  |  |  |  |  |  |  |  |  |  |  |
| **Tumor size (cm)** |  |  |  |  | **0.049*** | **P53 mutation (Y/N)** |  |  |  |  | 0.980 |
|  | ≥ 3 | 174 | 101 | 73 |  |  | Yes | 87 | 48 | 39 |  |
|  | < 3 | 33 | 13 | 20 |  |  | No | 120 | 66 | 54 |  |
| **Mavi (Y/N)** |  |  |  |  | 0.159 | **BCLC stage** |  |  |  |  | 0.919 |
|  | Yes | 22 | 9 | 13 |  |  | 0 & A | 172 | 95 | 77 |  |
|  | No | 185 | 105 | 80 |  |  | B & C | 35 | 19 | 16 |  |
| **Mivi (Y/N)** |  |  |  |  | 0.385 |  |  |  |  |  |  |
|  | Yes | 120 | 63 | 57 |  |  |  |  |  |  |  |
|  | No | 87 | 51 | 36 |  |  |  |  |  |  |  |
