# Supplementary material for: The novel miR-1269b-regulated protein SVEP1 induces hepatocellular carcinoma proliferation and metastasis likely through the PI3K/Akt pathway
Source: Cell Death Dis. 2020 May 5;11(5):320. doi: 10.1038/s41419-020-2535-8 (PMC7200779; doi:10.1038/s41419-020-2535-8)
Supplement: Supplementary file 10 — Supplementary table 5 [file 41419_2020_2535_MOESM10_ESM.docx]

**Table S5. miRNAs identified in high recurrence group**

| **miRNA** | **HN18244** | **HN19308** | **HN332390** | **HN350731** | **HT18244** | **HT19308** | **HT332390** | **HT350731** | **Fold Change** | **log2 Fold Change** | **pval** | **padj** |
| --- | --- | --- | --- | --- | --- | --- | --- | --- | --- | --- | --- | --- |
| hsa-miR-934 | 9.958712095 | 3.298508147 | 10.314213 | 13.02626978 | 0 | 0 | 0 | 0 | 0 | 0 | 1.13759E-05 | 0.001296857 |
| hsa-miR-10a-5p | 79587.81401 | 42119.75003 | 91943.47321 | 67994.68584 | 5379.962006 | 15719.09023 | 22748.77477 | 6458.041028 | 0.178613996 | -2.485082962 | 0.000453901 | 0.025872379 |
| hsa-miR-4661-5p | 1.106523566 | 2.199005431 | 2.578553249 | 0.814141861 | 50.69362943 | 86.36862766 | 49.14697377 | 4.767589875 | 28.51156033 | 4.833475089 | 0.000247074 | 0.018777615 |
| hsa-miR-4686 | 115.0784509 | 114.3482824 | 45.55444074 | 61.87478147 | 0.804660785 | 0 | 0 | 0 | 0.002388738 | -8.709535366 | 8.90068E-07 | 0.000202936 |
| hsa-miR-4679 | 40.94137194 | 16.49254073 | 49.85202949 | 28.49496515 | 1.609321569 | 5.398039229 | 3.137040879 | 0 | 0.074711547 | -3.742524949 | 0.000376006 | 0.024494111 |
| hsa-miR-490-5p | 8.852188529 | 6.597016294 | 6.876141998 | 6.513134892 | 0 | 0 | 1.045680293 | 0 | 0.036259894 | -4.785481478 | 0.000217275 | 0.018777615 |
| hsa-miR-802 | 5.53261783 | 8.796021725 | 0.85951775 | 4.070709307 | 0 | 0 | 0 | 0 | 0 | 0 | 0.000927929 | 0.047015066 |
| hsa-miR-199a-5p | 64430.65421 | 20240.74549 | 76784.15866 | 58775.3434 | 2338.34424 | 1417.884971 | 10175.51493 | 694.7059532 | 0.066414159 | -3.912365347 | 2.07258E-06 | 0.000315033 |
| hsa-miR-1269b | 7.745664963 | 42.88060591 | 7.735659748 | 4.070709307 | 4238.953013 | 2688.223536 | 7605.232771 | 1872.981737 | 262.7694596 | 8.037653798 | 1.38762E-08 | 6.32754E-06 |
