# Supplementary material for: The novel miR-1269b-regulated protein SVEP1 induces hepatocellular carcinoma proliferation and metastasis likely through the PI3K/Akt pathway
Source: Cell Death Dis. 2020 May 5;11(5):320. doi: 10.1038/s41419-020-2535-8 (PMC7200779; doi:10.1038/s41419-020-2535-8)
Supplement: Supplementary file 11 — Supplementary table 6 [file 41419_2020_2535_MOESM11_ESM.docx]

**Table S6. miRNAs identified in low recurrence group**

| **miRNA** | **HN18244** | **HN19308** | **HN332390** | **HN350731** | **HT18244** | **HT19308** | **HT332390** | **HT350731** | **Fold Change** | **log2 Fold Change** | **pval** | **padj** |
| --- | --- | --- | --- | --- | --- | --- | --- | --- | --- | --- | --- | --- |
| hsa-miR-873-5p | 2.437634989 | 6.864611712 | 5.168830389 | 16.02738611 | 16.02634687 | 0 | 1.304991289 | 0 | 3.03266789 | 0.837485035 | 0.111234075 | -3.168329289 |
| hsa-miR-122-5p | 1609810.49 | 1180195.794 | 1946600.908 | 1372573.326 | 1218724.735 | 622649.6959 | 671647.6966 | 1117440.775 | 613858.4818 | 406811.7057 | 0.468402393 | -1.094179649 |
| hsa-miR-934 | 18.28226242 | 14.58729989 | 9.045453181 | 4.006846527 | 10.09066284 | 0 | 0 | 0 | 1.010889297 | 0 | 0.018047558 | -5.792052541 |
| hsa-miR-3174 | 1.218817495 | 0 | 0 | 0 | 0 | 7.357602161 | 1.304991289 | 1.808648265 | 6.065335779 | 5.862395245 | 18.37762654 | 4.199878551 |
| hsa-miR-4686 | 69.47259719 | 46.33612905 | 67.19479506 | 78.13350728 | 89.62882877 | 0 | 0 | 1.808648265 | 0 | 1.67497007 | 0.009931464 | -6.653777945 |
| hsa-miR-490-5p | 9.750539957 | 5.148458784 | 15.50649117 | 5.008558159 | 8.309957634 | 1.051086023 | 0 | 3.61729653 | 0 | 0 | 0.106769324 | -3.227430884 |
| hsa-miR-135a-5p | 26.81398488 | 19.73575867 | 37.47402032 | 24.04107916 | 26.71057811 | 146.1009572 | 366.7025522 | 47.92917902 | 713.6878434 | 649.8883871 | 14.27789209 | 3.835711098 |
| hsa-miR-138-5p | 21.9387149 | 13.72922342 | 34.88960513 | 21.03594427 | 14.24564166 | 2.102172046 | 7.829947734 | 1.808648265 | 1.010889297 | 0 | 0.120481503 | -3.053116427 |
